# Mitochondrial Reactive Oxygen Species (ROS) Production Alters Sperm Quality

**DOI:** 10.3390/antiox10010092

**Published:** 2021-01-11

**Authors:** Rosanna Chianese, Riccardo Pierantoni

**Affiliations:** Dipartimento di Medicina Sperimentale, Università degli Studi della Campania Luigi Vanvitelli, via Costantinopoli 16, 80138 Napoli, Italy; riccardo.pierantoni@unicampania.it

**Keywords:** oxidative stress, sperm physiology, ROS impact on sperm quality

## Abstract

Besides ATP production, mitochondria are key organelles in several cellular functions, such as steroid hormone biosynthesis, calcium homoeostasis, intrinsic apoptotic pathway, and the generation of reactive oxygen species (ROS). Despite the loss of the majority of the cytoplasm occurring during spermiogenesis, mammalian sperm preserves a number of mitochondria that rearrange in a tubular structure at the level of the sperm flagellum midpiece. Although sperm mitochondria are destroyed inside the zygote, the integrity and the functionality of these organelles seem to be critical for fertilization and embryo development. The aim of this review was to discuss the impact of mitochondria-produced ROS at multiple levels in sperm: the genome, proteome, lipidome, epigenome. How diet, aging and environmental pollution may affect sperm quality and offspring health—by exacerbating oxidative stress—will be also described.

## 1. Mitochondria: A Central Role in Sperm Physiology

Mitochondria are classically known for being eukaryotic cell powerhouses due to their ability to produce ATP via oxidative phosphorylation [1]. They are highly dynamic organelles, able to adapt their shape to the physiological needs of the cell, suggesting their participation in numerous other physiological functions beyond ATP production.

In this regard, they create transient contacts with endoplasmic reticulum membranes and lysosomes, essential for autophagy, mitochondrial motility and fission, lipid and calcium (Ca^2+^) fluxes, [2,3] as well as glucose homeostasis and mitochondrial DNA (mtDNA) replication [4]. Mitochondrial Ca^2+^ uptake regulates cytosolic Ca^2+^ homeostasis, thus influencing extracellular Ca^2+^ entry [5,6]. Mitochondrial electron transfer chain also promotes reactive oxygen species (ROS) generation, besides its involvement in ATP synthesis. These molecules participate in both signalling pathways and in oxidative stress, if unbalanced produced [7]. Mitochondrial contribution to steroid hormone biosynthesis—by catalyzing the conversion of cholesterol to pregnenolone—has also been investigated [8].

The number, shape and structure of the mitochondria dramatically change during mammalian spermatogenesis, with secondary spermatocytes and spermatids that have more condensed mitochondria [9,10]. Despite the loss of the majority of the cytoplasm during spermatid differentiation, a number of mitochondria still remain in spermatozoa (SPZ), rearranging in tubular structures at the level of the midpiece of the flagellum [7]. During sperm maturation, mitochondria become more polarized in rodent species after epididymal maturation or wrapped in humans after capacitation [11].

Mitochondria’s role as energy provider is surely fundamental for sperm motility. In fact, defects in sperm mitochondrial ultrastructure are associated with decreased sperm motility and asthenozoospermia [12,13]. However, both metabolic pathways—glycolysis and mitochondrial oxidative phosphorylation—may sustain sperm motility, and several glycolytic enzymes are distributed in the sperm tail [14], thus suggesting a great versatility of SPZ in their metabolism by using glycolysis exclusively, mitochondrial oxidative phosphorylation or both as dual sources of energy according to the availability of substrates in the female genital tracts [15,16].

An important prerequisite to produce ATP is the maintenance of a positively charged membrane potential [17]. The treatment of human sperm with an oxidative uncoupler reduces mitochondrial membrane potential, impairing sperm motility and fertility [18]. Accordingly, low mitochondrial membrane potential and high ROS production have been detected in SPZ from infertile patients [19].

MtDNA is another candidate aspect strongly correlated with sperm physiology and quality. MtDNA has a loosely packaged structure and, therefore, it is more easily damaged by ROS than the nuclear genome [20]. Point mutations, rearrangement and/or decreased content of mtDNA are all features correlated with sperm dysfunctions and infertility [17,21]. Conversely, a low mtDNA copy number has been suggested as an indicator of good-quality sperm [22]; thus, its manipulation may be a powerful therapeutic strategy to decrease aging-associated mtDNA mutations [23]. Interestingly, even if still controversial, DNA methylation in mtDNA has been found to be associated with both transcriptional regulation and mtDNA copy number [24]. Such an epigenetic process takes part to the largely unexplored field of the mitochondrial epigenetics, together with the presence of non-coding RNAs inside the mitochondria.

Coding and non-coding RNAs have been widely analyzed as epigenetic regulators involved in the modulation of sperm functions [25,26,27,28,29]. In this regard, microRNAs (miRNAs), encoded by the nuclear or mitochondrial genome, have a dual role through the regulation of the nuclear genome, encoding mitochondria-related proteins, or translocating into the mitochondria in order to regulate mitochondrial genome expression [30]. MiRNAs might modulate sperm functions through mitochondria-dependent pathways; their aberrant expression in sperm of aging males has been correlated with poor semen quality caused by the suppression of the mitochondrial function and the reduction of ATP production [31]. Other small RNAs encoded by the mtDNA, overall known as mitosRNAs, have been recently discovered [32]. Interestingly, different isoforms of miRNAs derived from mtDNA have been found in oocytes, SPZ, and zygotes, with SPZ that show a predominance of the mito-miRNA isoform named paramiR, partially corresponding to the 5′ region of the canonical miRNA. Among mitosRNAs, mito-piRNAs are the most predominant mitosRNA population in the mitochondria of mouse germ cells. Both piRNAs and their associated proteins play a key role in mitochondrial homeostasis and nuclear communication [32]. MtDNA also encodes a set of long non-coding RNAs (lncRNAs) [33]. Intense crosstalk exists between mitochondria and nucleus; it is mediated by lncRNAs of nuclear origin, through molecular trafficking that is still an exciting issue to investigate. Once imported into the mitochondria, lncRNAs regulate mtDNA replication, RNA processing, hormone signalling, mitochondria-mediated apoptosis, and mitochondrial bioenergetics [34]. Mitochondria-encoded lncRNAs (mt-lncRNAs) have a different structure in comparison with the nuclear lncRNAs; they are chimeric, deriving from more than one gene with the merging of their transcripts as a post-transcriptional product via trans-splicing reactions [35]. A typical chimeric mt-lncRNA has been localized in the nucleus of mouse sperm, suggesting the export of mitochondrial material towards the nucleus. Conversely, limited evidence exists about circular RNAs (circRNAs) of mitochondrial origin (mt-circRNAs). This recently discovered class of non-coding RNAs plays critical roles in key physiological functions, working as microRNA (miRNA) sponges, protein scaffolds, and translation templates. Evidence in testis and SPZ correlates them with germ cell progression and sperm quality [36]. Gao et al. [37] found three mt-circRNAs by studying circRNAs expression in cattle testis, but their functions were not explored. CircRNAs whose host genes are derived from the mitochondrial genome have also been discovered in human testis [38] and SPZ [28], but, as in cattle, their potential role has not been thorough.

Although the characterization of the mitoRNA landscape in mouse male germ cells, gametes, and zygotes opens the door to novel mechanisms of regulation in mitochondria, much effort is required to unravel the biological functions of these RNAs in germ cell functions and how these molecules may coordinate signalling pathways between nucleus and mitochondria [39].

In the scenario of the mitochondria involvement in sperm physiology, proteomic studies have tried to identify dysfunctional mitochondrial proteins responsible for infertility [40,41]. Interestingly, a large percentage of these proteins—especially engaged in cell metabolism and energy production, protein folding/degradation, vesicle trafficking and cytoskeleton organization—are deregulated in low motile SPZ [40,42]. As the endoplasmic reticulum, mitochondria need dedicated protein-folding machinery in order to control the amount of unfolded or misfolded proteins produced under stress conditions [43,44]. Such machinery appears deregulated in the case of male infertility [45].

**Figure 1:**
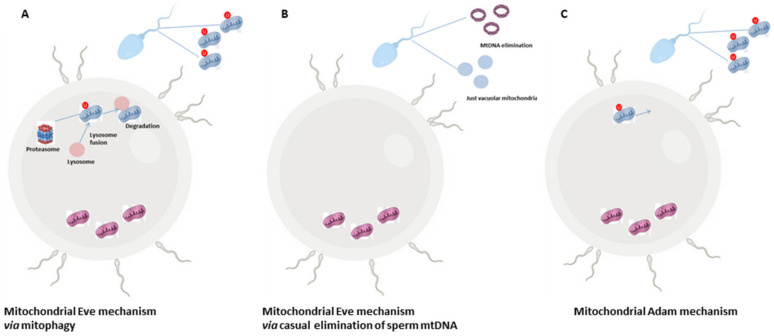
The Mitochondrial Eve or Adam mechanism. Most literature hypothesizes that the zygote exclusively inherits maternal mitochondria so that paternal ones that enter into the zygote are eliminated by the involvement of the proteasome-autophagy-lysosome pathway (**A**). A second hypothesis that still sustains maternal inheritance expects a precocious elimination of paternal mtDNA from motile SPZ and a change in mitochondria morphology creating vacuolar organelles in order to provide energy for sperm motility (**B**). A third hypothesis does not exclude the paternal inheritance of mitochondria with an uneven distribution of these organelles inside the embryo (**C**).

Another intriguing aspect concerns the fate of sperm-derived mitochondria during fertilization, since in most mammals, the sperm tail is also incorporated along with the sperm genome into the oocyte [46]. However, the selective elimination of paternal mitochondria from the zygote may be the result of a developmental pressure promoting the strictly maternal inheritance of mitochondria. Such a transmission is known as maternal inheritance [47,48] or cleverly nicknamed the paradigm of Mitochondrial Eve by Lewin (1987) [49]. The mechanism in support of the maternal inheritance of mitochondria includes an early modification of sperm mitochondria, already during spermatogenesis, through a pre-labelling with ubiquitin [50]. Into the zygote, ubiquitin-labelled sperm mitochondria are selectively recognized by the proteasome-dependent proteolytic machinery and then eliminated by lysosomes (Figure 1A) [51]. Actually, a cascade of events may be involved, with autophagy—referred to as “sperm mitophagy”—as an intermediate mechanism between ubiquitination and lysosome degradation [52] and the combined action of multiple proteins working, at least in higher mammals [48,53]. However, by using transgenic mouse strains, mitophagy has been excluded as the involved pathway in sperm mitochondrial degradation; rather, the elimination of sperm mtDNA in most motile SPZ before fertilization has been suggested as a passive casual event, at least in mice, that leaves in cells just vacuolar mitochondria—deprived of mtDNA—in order to supply the amount of energy necessary for fertilization (Figure 1B) [54]. Such evidence does not exclude that—in rare cases—some cells and tissues could inherit paternal mtDNA, known as the Mitochondrial Adam mechanism, with an uneven distribution of mitochondria (Figure 1C). However, since human eggs contain more than 100.000 copies of mtDNA in comparison with sperm that just contains 100 copies, a possible dilution effect has also been hypothesized. Interestingly, in cases of diseases caused by mtDNA mutations, the coexistence of normal and mutant mtDNA molecules in a single cell—a situation called heteroplasmy [55]—not only contributes to the disease severity, but it could not be explained just by maternal inheritance, thus suggesting that paternal mtDNA could be passed to the offspring [56].

Although the central dogma of maternal inheritance of mtDNA still remains, the potential impact of paternal mtDNA on embryo development cannot be ignored [54,57,58].

## 2. Mitochondria: Key Producers of ROS. A Focus

ROS generation requires the activation of the mitochondrial electron transport chain and mainly takes place on the inner mitochondrial membrane during the process of oxidative phosphorylation [59]. This essential cellular process involves five big protein complexes that, in succession, transfer electrons donated from nicotine adenine dinucleotide (NADH) to O_2_. Meanwhile, mitochondrial membrane potential is created through an active pumping of positively charged protons (H^+^) from the mitochondrial matrix into the intermembrane space; in this way, when protons re-enter in the mitochondrial matrix through the enzymatic complex V, there is the generation of a proton-motive force that allows it to generate ATP [60]. Under stress conditions or by accident, the electron transfer along the mitochondrial electron transport chain may not be perfect, with the leakage of electrons and the partial reduction of oxygen to form superoxide anion (O_2_^−^) as a consequence. Such an anion can be thrown towards the mitochondrial matrix from complex I and towards both the intermembrane space and mitochondrial matrix from complex III [61]. Subsequently, two dismutases (SOD enzymes) quickly dismutate the superoxide anion to hydrogen peroxide (H_2_O_2_) in the mitochondrial intermembrane space. Afterwards, H_2_O_2_ is fully reduced to water by glutathione peroxidase (GPX). However, both O_2_^−.^ and H_2_O_2_, generated in this process, are considered as mitochondrial ROS. In addition, O_2_^−.^ can undergo a radical-radical reaction with nitric oxide (NO) to form peroxynitrite (ONOO_2_^.−^). While O_2_^−.^ is not considered a good candidate as a signalling transduction molecule because it has electrophilic properties and short half-life and can hardly pass through the mitochondrial outer membrane, H_2_O_2_ is electrophobic and more stable; thus, its concentration inside the mitochondria is 100 times greater than that of O_2_^−.^ [62].

Mitochondrial ROS are highly reactive and toxic molecules so that mammalian cells need a number of antioxidant enzyme systems to scavenge them. Usually, after SOD action, H_2_O_2_ is quickly reduced to water by two other enzymes, catalase (CAT) and GPX. All these mitochondrial antioxidant enzymes are encoded by the nuclear genome and need to be imported into the mitochondria after their synthesis in the cytoplasm. The action of the antioxidant enzymes is surely corroborated by several natural antioxidants such as vitamin E, whose effectiveness is, however, limited since it is not able to accumulate within mitochondria. The development of synthetic mitochondrial ROS scavengers able to easily pass through all biological membranes has been a useful instrument for addressing this issue [63].

In mitochondria, ROS generation is strictly regulated by several factors. First of all, the mitochondrial membrane potential: a higher, more polarized potential has been widely associated with greater mitochondrial ROS generation [61], and the metabolic state of mitochondria—measured in terms of ATP synthesis—modulates the endogenous production of ROS. Also converging in such a direction are sirtuins, NAD^+^-dependent deacetylases able to counteract the overproduction of ROS via epigenetic modifications [64]. They are finely localized among the nucleus, the cytosol, and the mitochondria, and are activated by resveratrol, an antioxidant polyphenol compound isolated from grape skins. Among the seven members of the sirtuin family, a prominent role is played by Sirt1, whose activity is deeply impaired by oxidative stress, suggesting a crosstalk between Sirt1 function and ROS signalling [65]. Furthermore, the potential ability of Sirt1 to counteract oxidative stress has also been investigated in the testis as a consequence of exposure to environmental contaminants [66].

What is clear is that, once thought as merely the by-products of cellular metabolism, nowadays mitochondrial ROS are deeply investigated as important signalling molecules. High ROS levels signal in cells, especially by promoting the oxidation of protein targets, thus triggering apoptosis/autophagy pathways and causing cell death as the final consequence [67].

## 3. Mitochondrial ROS and Sperm Quality

As previously described, aerobic cells physiologically produce ROS, such as hydroxyl radicals (•OH), O_2_^−.^, H_2_O_2_, NO, and so on, as obligatory metabolic products. Antioxidant systems—including enzymes such as superoxide dismutase (SOD), CAT, glutathione peroxidases (GPXs), thioredoxins (TRXs), and peroxiredoxins (PRDXs)—are charged with keeping ROS at low levels in cells [68].

The testis has developed a sophisticated array of enzymatic antioxidant systems [69,70], but also it relies on small non-enzymatic factors that work as free radical scavengers, such as: zinc—a core constituent of SOD, able to counteract lipid peroxidation [71]; vitamin C—especially produced by Sertoli cells and pachytene spermatocytes, whose deficiency leads to oxidative stress in testis [72]; and the pineal hormone melatonin, able to readily cross the blood-testes barrier to protect the germinal epithelium against oxidative stress.

An exacerbated production of ROS levels—known as oxidative stress—due to an overproduction of ROS and/or a dysregulation of the antioxidant scavenging system, becomes harmful in cells [73].

Given the complicated and dynamic sequence of events occurring during spermatogenesis (mitosis, meiosis and cell differentiation), with control systems required at both central and peripheral levels [74,75], a copious amount of ROS is physiologically generated by germ cells as by-products of their metabolism [76]. However, a moderate quantity of ROS is also convenient for regular functions, such as cell signalling, homeostasis, sperm capacitation, and sperm-egg interaction [77,78,79]. In particular, sperm capacitation is benefited by ROS mediation in cAMP generation, sperm plasma membrane cholesterol efflux, and tyrosine phosphatase activity inhibition. Conversely, the accumulation of ROS in the testis induces morphological alterations in the seminiferous epithelium [80] and cytoplasmic vacuolizations in both germinal and Sertoli cells [80] and apoptosis [81].

Multiple levels of the structural organization of sperm cells may be threatened by ROS: genome, epigenome, proteome, lipidome. All these aspects will be discussed in the following paragraphs.

### 3.1. Impact of Mitochondrial ROS on Sperm Genome and Epigenome

Among germ cells, SPZ are highly susceptible cells to oxidative insults; in fact, ROS-mediated damage to both the structural and functional integrity of SPZ is one of the major contributors to male infertility. The outcome of a pregnancy, as well as the health trajectories of the offspring, are negatively impacted by damaged or defective SPZ [82,83].

It is well known that during spermiogenesis, spermatids drastically change the folding of their genome, replacing histones with transition proteins first and protamines later [36,84]. Alternatively, transition proteins do not displace histones, but rather drive the recruitment and processing of protamines, which are themselves responsible for histone eviction, thus suggesting a cooperation between transition proteins and protamines, instead of a consequential activity [85]. However, although the majority of the sperm genome is bound to protamines, a small percentage (~5–10%) of DNA is still organized in nucleosomes by residual histones, intriguingly containing telomeres and promoters of genes involved in early embryonic development [86]. This genomic compartment is particularly vulnerable to oxidative stress [83]. Moreover, in mice, the sperm nucleus shows a regionalized sensitiveness to oxidative DNA alterations, with peripheral and basal nuclear regions—this last one localized close to the midpiece—that are more sensitive [87]. Since there is non-random localization of chromosomes into the sperm nucleus and the notion of chromosomal territories [88], it is logical to find some autosomes, such as Chr19, Chr18 and Chr17, highly vulnerable to oxidative damage [89]. Conversely, sex chromosomes appear to be particularly well-protected [90].

Oxidative DNA damage in SPZ includes DNA fragmentation by single-strand and double-strand breaks, the introduction of abasic sites, such as O^6^-methylguanine, or oxidated bases, such as the 8-hydroxy-2′-deoxyguanosine (8-OHdG)—one of the main products of DNA oxidation, purine, pyrimidine and deoxyribose modifications, DNA-protein cross-linking with gene transcription arrested or inducted, as a consequence [91]. These effects are certainly compounded by the physical architecture of SPZ; since they suffer from the lack of essential cytoplasmic enzymes or a fully functional DNA repair system, the inability of the transcriptional activation of genes encoding the involved antioxidant enzymes, and the protection of their nuclear DNA by the entering of nucleases. What is alarming is that SPZ with damaged DNA are still able to fertilize, with dangerous implications for the embryo. Increased oxidative DNA damage in SPZ has a strong impact on next generations; it has been correlated with childhood cancers [92], brain disorders such as autism and schizophrenia [93], and so on.

Oxidative stress also affects epigenetic marks [94]. The presence of DNA base adducts, such as the 8-OH-dG, in CpG islands alters the interaction between DNA and DNA methyltransferases, preventing the adjacent cytosine methylation and leading to a global hypomethylation which is associated with Sertoli cell-only syndrome, testis cancer, and hypospermatogenesis in humans [95,96]. After fertilization, conventional methylcytosine (mC) undergoes oxidation in 5-hydroxymethylcytosine (5HmC) via the action of the Ten-Eleven Translocation (TET) enzymes. This chemical modification is the starting point for active demethylation of paternal chromatin [97]. Post-testicular oxidative alterations of SPZ may generate an excessive production of 5HmC that changes the kinetics of paternal DNA demethylation influencing the embryo development. The oxidation can also affect DNA methyltransferase activity itself, thus decreasing DNA methylation [98].

Paternal histones and protamines are also targets of oxidative stress, as will be explained below, with potential hazardous effects on the embryo development and the health of future generations. As a part of the epigenetic signature of sperm cells, the non-coding RNA payload is gaining attention. Interestingly, along the epididymis, sperm non-coding RNA profile dynamically changes as a consequence of the epididymal epithelial cell secretion, via epididymosomes and/or in stress conditions [99]. A useful animal model to shed light on the effect of the oxidative stress on sperm non-coding RNA payload is the *GPX5* knockout mouse, whose epididymal epithelium has a decreased piRNA content [100].

### 3.2. Impact of ROS on Sperm Lipids and Proteins

Beyond the genome and epigenome, numerous other macromolecules carried by SPZ are in the crosshairs of oxidative stress. These are lipids and proteins.

Sperm fragility to ROS is, in fact, aggravated by a very peculiar lipid composition of its plasma membrane that—in comparison to all the other differentiated cells—is richer in polyunsaturated fatty acids (PUFAs, [101]), a class of particularly vulnerable lipids whose peroxidation affects membrane fluidity and permeability, important properties for both flagellar movements and fusion with the vitelline membrane of the oocyte [102]. As a key target of ROS, the sperm plasma membrane can stimulate a downstream signal cascade, damaging both nuclear and mitochondrial genomes.

The involved organelles are, therefore, mitochondria: they are both source and targets of ROS. Antioxidant system dysregulation alters mitochondria membrane potential with higher and lower production of free radicals and ATP, respectively [103], which in turn can trigger lipid peroxidation [82]. In germ cells, mitochondria dysfunction implies meiotic arrest, whereas in SPZ this means disorganization of the axonemal apparatus required for sperm motility and asthenozoospermia as a consequence [104]. Sperm motility is also damaged by thiol oxidation of the *α*-tubulin protein, a structural component of the sperm flagellum that impairs microtubule polymerization [105]. In this regard, the first observation that—under high oxygen tension conditions—human SPZ lose their motility dates back to 1943, with studies by MacLeod et al. [106]. Another important aspect linked to sperm mitochondria is their genome, not compacted by protamines and thus more vulnerable to oxidative attacks [89]. Considering that the most ascertained hypothesis describes that paternal mitochondria are quickly destroyed after fertilization to make way for the maternal mitochondria, oxidative damage to mtDNA may not be relevant for embryo development. However, as previously described, some evidence does not exclude a potential paternal inheritance of mitochondria. In that case, a damaged paternal mtDNA may be involved in several pathological processes inside the embryo or future generations.

Together with lipids, sperm proteins, especially localized in the nucleus, can be affected by ROS through carbonylation and redox thiol modification [107]. Sperm nuclear proteins that contain thiols are especially protamines whose oxidation completely alters chromatin folding and function. Although protamine change is not expected to be damaging to the embryo considering their quick removal after fertilization, it is plausible that protamine carbonylation affects protein–protein cross-linking and the global nucleus architecture [108]. More dangerous for the embryo is the oxidation of paternal histones that still remain after fertilization, creating unsuspected problems in the developing embryos. In this regard, oxidative stress increases histone methylation, correlated with double-stranded breaks and poor sperm quality. Together with methylation, histone acetylation is also impaired by oxidative stress [109]. Chromatin remodelling is unavoidably compromised.

Several other protein modifications can be promoted by ROS in SPZ. S-nitrosylation generally affects enzymes involved in ATP production and ion channels; tyrosine (Tyr) nitration alters sperm protein function leading to physiological or pathological effects, depending on the protein target and the level of ROS generated. Enzymes involved in glycolysis and Krebs cycle are especially impaired by ROS through a Tyr nitration modification [110]. As a direct consequence, ATP production is severely diminished and sperm motility impaired. Concerning sperm motility and beyond thiol oxidation, α-tubulin may also be modified by Tyr nitration, thus to interfere with the appropriate microtubule polymerization in the sperm flagellum. Sperm capacitation is also associated with Tyr nitration. All these redox modifications of sperm proteins are mechanisms by which ROS control cell signalling, stimulating or inhibiting the activity of proteins involved in a large variety of processes linked to sperm physiology [110].

It is clear that sperm cells are both vulnerable to ROS and good producers of ROS, especially at the onset of capacitation. Under stress conditions and as a result of membranous lipid peroxidation, SPZ generate cytotoxic lipid aldehydes such as malondialdehyde (MDA) and, above all, 4-hydroxynonenal (4-HNE; [111]). These molecules, in turn, stress ROS production, interfering with mitochondria activity and stimulating inflammation.

## 4. The Epididymis: The Microenvironment Orchestrating the Antioxidant Defences

SPZ retrieved directly from the testes are epigenetically immature, whereas along the epididymis they gain epigenetic maturity, but also accumulate oxidative damage. However, the epididymal epithelium physiologically protects SPZ against oxidative damage, through a battery of antioxidant enzymes [112]. In particular, among GPX enzymes, the isoform GPX5 is directly secreted by the epithelium of the *caput* epididymis, at the level of principal cells. *GPX5* knockout mouse produces SPZ with higher levels of DNA oxidation, compared to wild-type, suggesting the important role played by this enzyme in protecting SPZ from oxidative damage [113]. GPX enzymes cooperate with PRDXs to protect SPZ during their epididymal maturation [114]. PRDXs are differentially expressed from *caput* to *cauda* epididymis, in all epithelial epididymal cells, except in clear cells; under stress conditions, SPZ collected from *cauda* epididymis are impaired because of membrane lipid peroxidation, DNA oxidation and lower motility and both GPXs and PRDXs are downregulated. Interestingly, this protective enzymatic battery can be achieved by SPZ along the epididymis via epididymosomes [114].

However, as previously underlined, oxidative processes physiologically contribute to sperm cell maturation.

This positive action of ROS has been deeply characterized just along the epididymis, where post-testicular maturation of SPZ takes place [115]. There, sperm proteins undergo an impressive disulphide bridging [116,117]. Interestingly, in the *caput* epididymis, thiol groups carried by sperm proteins—located on both plasma membrane and intracellular organelles—are mainly free, instead in the *cauda* epididymis, most free thiols are converted into disulfide bridges, involved in protein–protein interactions and required for sperm motility. H_2_O_2_ is the oxidizing agent used from the disulphide isomerase enzyme. Among sperm proteins, protamines are especially oxidized to disulphide bonds. During epididymal transit, several protamine thiol groups are converted into disulfide bridges, which stiffen toroid organization and enclose the sperm nucleus in an optimal condensed state, but many other thiol groups remain free to mitigate oxidative attacks by blocking ROS [118]. Therefore, the anti-ROS action of protamines appears to be essential along the epididymis.

Additionally, the gradual increase in sperm motility that characterizes sperm maturation unavoidably generates ROS via the mitochondrial respiratory chain [119]. A probable evolutionarily trick to dampen the harmful effects of ROS has been to group mitochondria in the midpiece of flagellum, a very small and well-defined subcellular compartment where free radicals can be easily neutralized.

Therefore, the epididymis represents the most protective microenvironment from oxidative attack for SPZ, since there, SPZ that are transcriptionally and translationally silent do not possess instruments to counteract the harmful actions of ROS and are in the process of maturing in order to fertilize oocytes. Their only response along the epididymis—if faced with an excess of ROS—may be the apoptosis [120].

In both epididymal and seminal fluids—very promising sources of biomarkers of male infertility [121]—the more compelling need to enhance antioxidant ability is satisfied by an increase in non-enzymatic antioxidant molecules such as vitamins, polyamines, carnitine, and trace elements such as selenium [122].

In seminal plasma, ROS sources can be classified into endogenous and exogenous [123]. To the first group belongs immature SPZ and leukocytes. Immature SPZ—which have failed to complete normal morphological differentiation—have an excess of cytoplasm in their midpiece and contain the enzyme glucose-6-phosphate dehydrogenase involved in NADPH production. ROS production, fuelled by NADPH [124], takes place in two main compartments: the sperm membrane and mitochondria [125]. Inflammation or infection of the reproductive tract cause leukocytospermia: an increase in leukocyte number in the seminal plasma. These cells are a second endogenous source of ROS in the seminal plasma [126].

As will be further discussed, several extrinsic factors can induce oxidative stress impairing sperm quality. Special attention will be focused on diet, aging, and environmental pollution.

## 5. Diet, Aging and Environmental Pollution Damage Sperm Quality via Oxidative Stress and Alter the Health Trajectories of Future Generations

**Figure 2:**
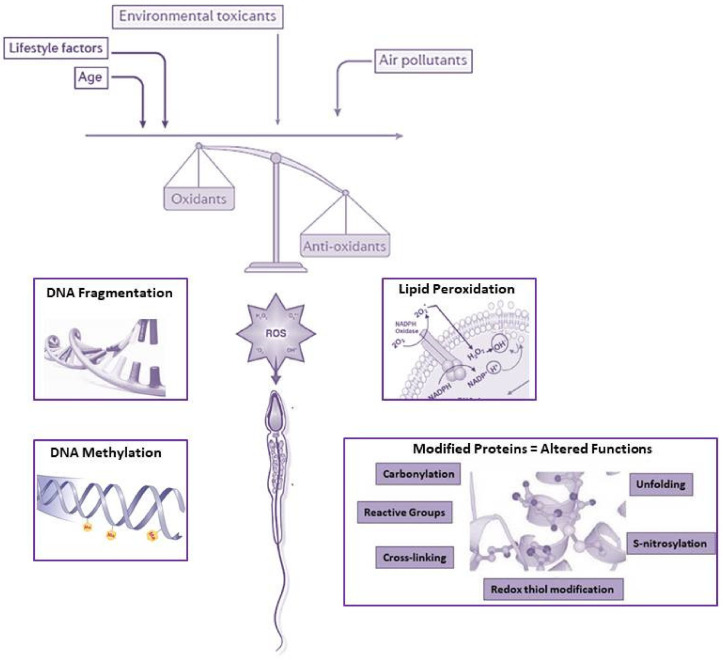
The impact of ROS on sperm quality at multiple levels. A drafting view of the main exogenous insults and the dangerous effects on sperm cells.

In addition to endogenous influences, a wide range of exogenous factors—including environmental and lifestyle-related factors—impact on sperm quality, causing male infertility [127,128,129,130,131,132]. All these lead to oxidative modifications of crucial components of SPZ (DNA, proteins, lipids), therefore altering their vital physiological functions [110], as summarized in Figure 2.

### 5.1. Diet

The obesity rate—especially promoted by dietary lifestyle—has registered a large increase in the last decades, mostly in developed countries. One of the negative health consequences of obesity is a reduced male fertility [133]. Within the testicular milieu, mitochondrial functioning has to support spermatogenesis progression in order to guarantee the production of good quality SPZ; a high-energy diet creates a suboptimal bio-energetic status in testis, just impairing mitochondrial function and DNA content [134]. As a consequence of oxidative stress, the antioxidant capacity significantly decreases within the testicular environment because of high-energy intake, especially impairing the activity of the proliferators-activated receptor γ coactivator1α (PGC-1α) and sirtuin 3 (SIRT3) [135]. Obese or overweight men have, in fact, lower sperm count and motility, higher DNA damage, and an altered sperm proteome involved in biological processes such as inflammation, translation, DNA damage repair and sperm functions [136,137]. ROS production in the testis and sperm is also exacerbated by the chronic inflammation generated by obesity. The high-fat concentration in obese gonads increases the internal temperature, aggravating oxidative stress and depleting antioxidant defence, thus modifying sperm parameters [136,138].

Interestingly, in mice, paternal obesity negatively leads to infertility and fat-related metabolic pathologies in the male offspring [139,140]. A suggested mechanism for this transgenerational inheritance of paternal acquired obesity may involve the epigenetic route. The methylation rate at several imprinted genes is significantly lower in sperm from overweight and obese men [141] and the histone composition at specific genes implicated in the development and cell fate decision is modulated by paternal obesity [142]. Small non-coding RNAs have also been involved in such inheritance, as evidenced by deep-sequencing analysis of testicular RNAs from high-fat diet mice showing several deregulated classes of RNAs, including miRNAs, piRNAs and fragments of tRNAs [143]; these last molecules are known to significantly contribute to intergenerational inheritance of metabolic disorders [99,144].

As previously outlined, several dietary non-enzymatic factors/micronutrients such as zinc, selenium, lycopene, vitamins E and C, glutathione, resveratrol, melatonin, and albumin—as small molecules capable of trapping free radicals—are able to fight against oxidative attack in testis, improving sperm concentration and motility in infertile obese men [145]. In particular, vitamin C transporters—localized in Sertoli cells—play a fundamental role for the normal delivery of vitamin C to germ cells in the adluminal compartment of seminiferous tubules [72] to further protect and control testis development and differentiation [146]. Plants are rich in active substances with chemical groups including saponin, phytosterols, and carotenoids, whose oral intake prevents lipid peroxidation and reduces ROS production, thus decreasing the risk of infertility [147]. Dietary supplementation with docosahexaenoic acid (DHA) improves seminal antioxidant status and sperm quality [148,149]. An interesting example is curcumin, a Chinese herb monomer with antioxidant and anti-inflammatory properties which is able to improve the sperm motility in patients with leukocytospermia [150].

All this evidence clearly supports the oxidative stress as an intermediate negative link between obesity and sperm quality.

### 5.2. Aging

Fertility in women declines with age; instead, men produce SPZ throughout their life. However, advanced age in men is associated with a decline in steroidogenesis [151] and sperm quality, as a consequence of an increased testicular oxidative stress that generates mutations in both nuclear and mitochondrial genome, reduction in DNA replication fidelity and inefficiency in DNA repair [152]. Sperm chromatin integrity is deeply impaired during the aging [153]; such an effect is more exacerbated in the absence of *PRDX6*, suggesting the protective role of this enzyme in age-associated decline in the sperm quality and fertility in mice [154].

Brown Norway old male rats are an excellent model to evaluate the effects of aging on sperm quality. Interestingly, this animal model produces SPZ with altered chromatin and an impressive decrease in the antioxidant enzymatic activity along the epididymis [155]. Furthermore, the isolation of germ cells from aging rodents has allowed us to highlight that the antioxidants SOD1 and CAT play a critical, but not equivalent, role in the response to oxidative stress during aging [156]. In fact, germ cells from aged mice lacking *SOD1* display increased ROS levels and greater susceptibility to DNA damage in comparison with aged mice lacking *CAT* that, instead, display compensatory antioxidant mechanisms [157].

The dysfunction of the blood-epididymis barrier and the accumulation of damaged epithelial cells has also been investigated, with the evidence of an induced active response of immune cells. As a consequence, the epididymal duct is invaded by an increased number of leukocytes—one of the major endogenous ROS producers in reproductive tracts—thus contributing to the overall increase in ROS levels in the semen [158]. Likewise, the antioxidant protection in the epididymis becomes inefficient: SOD and GPX levels drastically decrease as advancing in age [159].

A typical biomarker of chronological aging is telomere length that is maximum at birth and progressively decreases with advancing age as a result of combined effects of oxidative stress, inflammation, and repeated cell replication on it [160]. In comparison with somatic cells, sperm telomere length surprisingly increases in older men as a kind of a biological resistance against the aging; furthermore, such elongation is translated into longer leukocyte telomere length in the offspring [161,162]. However, the involved molecular mechanism is still under investigation.

The devastating effects of impaired sperm DNA integrity on early embryonic development have been undoubtedly evaluated [163]. Interestingly, in humans, germ line de novo mutations are observed in the offspring as a direct consequence of father’s age, at the moment of conception [164]; accordingly, offspring also shows an increased susceptibility for diseases such as schizophrenia, autism, myotonic dystrophy, Huntington disease, and childhood cancers [165]. The father’s advanced age also correlates with abnormal sperm DNA methylation that is not only confined to differentially methylated regions, but widespread in the genome, especially at the level of regions associated with the control of schizophrenia and/or bipolar disorders [166]. Interestingly, the methylation of ribosomal DNA increases with age in both somatic and sperm cells, thus influencing nucleolar formation and embryo development [167].

However, aging is associated with widespread epigenetic changes in sperm cells. MiRNAs are typical mediators of their epigenetic regulation; besides, they are able to induce mitochondrial dysfunction and increase ROS production. Accordingly, miRNA content in the seminal plasma significantly changes with age [168]. By performing high-throughput sequencing of small RNAs in sperm, oocytes and embryos of aged and young mice, it has been demonstrated that there is a differential expression of numerous miRNAs and piRNAs in correlation with the age, most of them involved in embryo development [169]. A typical example of such a deregulation concerns miR-574, upregulated in the sperm of older mice and significantly related to a decreased sperm motility. Interestingly, miR-574 has been shown to suppress mitochondrial function and ATP production by directly targeting the *mt-ND5* gene, a typical mitochondrial gene encoding NADH dehydrogenase 5, an essential component of the complex I [169].

Although the negative effects correlated with increasing paternal age have been analyzed in several studies, the detailed molecular mechanisms hampering sperm functions are still poorly understood.

### 5.3. Environmental Pollution

The negative effects of environmental factors on sperm functions firstly reflect the inhibition of both gametogenesis and steroidogenesis as a consequence of a disruption of the hypothalamo-pituitary-gonadal axis, considering their ability to mimic estrogens/androgens [131,170]. In addition, alterations in the hormonal milieu contribute to induce oxidative DNA damage with double or single-stranded breaks, as well as epigenetic modifications of sperm cells [128,130,132]. Parabens, phthalate esters, and bisphenols are able to induce oxidative stress by virtue of their ability to activate ROS generation, decrease enzymatic and non-enzymatic antioxidants in both animal models and seminal plasma of infertile patients, and affect membrane lipids [171,172,173]. In particular, Bisphenol A (BPA)-induced oxidative stress is associated with a loss of sperm motility, reduced viability, premature acrosome reaction and alteration in sperm proteome. BPA also increases lipid peroxidation in sperm, thus affecting its ability for fertilization [130,172,174,175]. These effects may be mediated by oxidative-apoptotic mechanisms, since BPA is able to reduce mitochondrial membrane potential, promote ROS generation and DNA fragmentation in the sperm of several species [176]. In vitro effects of BPA have also been studied on human motile SPZ whose exposure to scalar concentrations of BPA produces a decrease of the mitochondrial membrane potential, accompanied by mitochondrial superoxide anion generation, activation of caspase-9 and caspase-3 and a significant decrease in motility, as final effect [177]. However, inconclusive findings about the possible adverse impact of BPA exposure on male fertility arising from clinical studies have also been discussed [176]. With similar oxidative mechanisms, BPF and BPS—alternative molecules to BPA—are also able to disrupt reproductive functions [130,178]. Plastics and endocrine disruptors then regulate the epigenetic signature of sperm cells, generating in them an anomalous state of DNA methylation, altering the sperm histone code as well as miRNA profiling [132,179].

Maternal exposure to environmentally relevant doses of BPA causes reproductive dysfunction in F1 adult male rats, through intergenerational inheritance mechanisms. Testicular oxidative stress is cause of interstitial necrosis and germinal cell degeneration; however, testicular damage can be mitigated by the co-treatment with melatonin, a potent antioxidant [180]. Accordingly, a prolonged exposure of dams, during all gestational periods, impairs spermatogenesis in progenies by decreasing antioxidant defence and Sirt1 expression, a key sensor of ROS production [66].

Through the sperm genome and epigenome, paternal exposure to toxicants has impacts on progeny outcome, with an increase in pre- and post-implantation loss, external malformations and altered behaviour, in subsequent generations [181]. In this regard, along generations, the epigenetic signature can undergo alterations, known as epimutations [132]. Sperm accumulates epimutations and epigenetically transfers to the offspring oxidative stress-induced molecular modifications, especially via DNA methylation, as a consequence of the environmental pollution [132,182].

## 6. Conclusions

Mitochondria functionality has a strong impact on the quality of sperm cells. First of all, they play the fundamental role to provide energy, even if the latest evidence suggests that glycolytic pathways may be equally useful to support sperm motility, with dependence on the availability of substrates in the female genital tracts. Point mutations, rearrangement and/or decreased content of mtDNA are all features correlated with low quality of sperm. Several small RNAs encoded by the mtDNA mediate an intense crosstalk between mitochondria and nucleus. The proteomic landscape of mitochondria is also deregulated in case of male infertility. Among all mitochondrial specializations linked to sperm physiology, the ability to produce ROS has been deeply investigated.

Sperm functions physiologically need to ROS. A balance between ROS levels and antioxidant defence creates the optimal state for cellular functions to be performed. When this balance is perturbed, a state of oxidative stress is created. Oxidative stress clearly harms SPZ at multiple levels: genomic, epigenomic, lipidomic, and proteomic, thus to be one of the major components of the male infertility landscape.

A wide spectrum of exogenous factors, such as inadequate dietary habits and environmental pollution, participates in exacerbating oxidative stress in sperm cells. What is more alarming is that oxidative stress not only represents the mechanism linking extrinsic factors to fertility, but also the mechanism by which paternal experience may influence the embryo development as well as the health of the offspring through the paternal transgenerational inheritance.
